# Exploring CP Array Antenna Characteristics and Its Applications to a Deployable Structure

**DOI:** 10.3390/s25092782

**Published:** 2025-04-28

**Authors:** Tae-Hak Lee, Kyoung-Joo Lee, WonSeob Lim, Sang-Hwa Yi

**Affiliations:** 1 Department of Electrical and Electronic Engineering, Yuhan University, 590, Gyeongin-ro, Bucheon-si 14780, Republic of Korea; 2 Electrical Apparatus Research Division, Korea Electrotechnology Research Institute (KERI), Ansan 15588, Republic of Korea; kynjlee@keri.re.kr; 3 Power Grid Research Division, Korea Electrotechnology Research Institute (KERI), Ansan 15588, Republic of Korea; skysyub116@keri.re.kr (W.L.); shyi@keri.re.kr (S.-H.Y.)

**Keywords:** array antenna, circular polarization, wireless power transmission, C-band, CubeSat, deployable antenna

## Abstract

In this article, we investigated the performance of circularly polarized (CP) array antennas for wireless power transmission (WPT) application. During the development process, three different mounting configurations for the transmitting CP antenna are considered, assuming that the proposed CP antenna could be a part of the WPT system. The wireless power transmission system employing the proposed antenna design as the transmitting antenna is intended for power transfer either between satellites in space or between a satellite and a ground segment. To implement the various mounting configurations, multiple transmitting array antennas, designed to operate at a center frequency of 5.8 GHz, were fabricated and tested. The performance of the proposed CP antenna was evaluated for each configuration in terms of return loss, gain, axial ratio, and radiation pattern. The experimental results of all three configurations support the design theory very well.

## 1. Introduction

Circularly polarized (CP) antennas play a critical role in modern communication systems due to their capability of minimizing polarization mismatch and maintaining signal quality. Unlike linearly polarized (LP) antennas, which require precise alignment between transmitting and receiving antennas, CP antennas provide realiable performance by accommodating various relative orientations between the two. This makes them indispensable in applications such as satellite communications, global navigation satellite systems (GNSS), and unmanned aerial vehicles (UAVs), where maintaining consistent communications despite movement is critical [1,2,3].

In addition to the above-mentioned conventional communication applications, CP antennas are particularly advantageous in wireless power transmission (WPT) systems. In WPT, efficient energy transfer depends on maintaining a stable link between the transmitter and receiver, even when the receiver’s orientation varies due to movement or deployment constraints. CP antennas address this challenge by ensuring that the received power remains relatively constant regardless of polarization alignment. This feature of CP wave propagation is especially beneficial for space-based WPT systems, where transmitting power to a CubeSat or other spacecraft demands resilience to unpredictable rotations and misalignments [4,5]. In [6,7,8,9,10], various resonator-type-based CP array antennas are reported. The PIN diodes are implemented to achieve the polarization reconfigurable characteristic in [6]. The multilayered substrate-integrated resonators are sequentially fed and realize more than 20% in axial ratio (AR) bandwidth at 60 GHz of frequency band [7,8]. In addition, three-dimensional microstrip-based patches or dielectric resonators are fed by microstrip line structure for the required AR bandwidth characteristics. However, in this manuscript, such circularly polarized propagation characteristics can be achieved using a simple microstrip-patch-based resonator and the sequentially rotated microstrip feed line structures.

**Figure 1:**
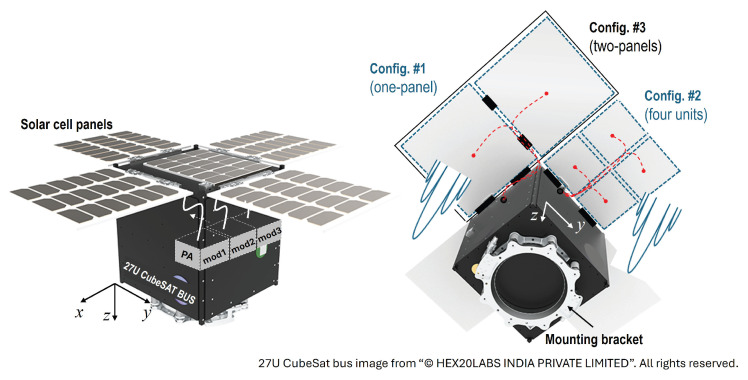
27U CubeSat example configuration.

Figure 1 illustrates an example of 27U CubeSat bus with the four panels of a solar cell and three different possible mounting configurations for WPT application. Considering the gain and efficiency characteristics of the transmitting antenna, both the array and panel sizes of the proposed design are optimized for integration with the 27U CubeSat bus. Please note that the example photograph of the CubeSat bus is sourced from Hex20 and the satellite platform and its electronics subsystems are commercially available from [11]. When the output signal from a power amplifier (PA) module placed in the satellite bus is applied to the transmitting antenna, the interface between them should be taken care of as indicated by the solid white lines in the left image of Figure 1. In our approach, the proposed antennas are assumed to be stacked beneath the solar cell panels, providing greater flexibility for both WPT scenarios: satellite-to-satellite power transmission and satellite-to-ground power transmission. Based on this assumption, we propose three different applicable mounting configurations, as shown in the right image of Figure 1. In the first configuration, a single 8 × 8 CP antenna array panel is placed beneath the solar cell panels. The second configuration divides the 8 × 8 array into four separate units, each with an independent input cable connected to PA modules. The third configuration combines two 8 × 8 array panels using deployable hinges. In this study, the CP array antennas for these mounting configurations were designed and their performance characteristics were evaluated.

In the following sections, we briefly introduce the PA module and its characteristic, which were designed by KERI for testing the WPT system performance in both ground and airborne environments. Subsequently, we provide a detailed designs of the CP array antenna. The performance of the proposed transmitting antenna is then evaluated under three different mounting configurations.

## 2. Design and Experimental Results

### 2.1. Power Amplifier Module Design

Before proceeding with the detailed antenna design, it is crucial to clarify the input signal characteristics from the PA modules, as they directly influence the interface requirements of the transmitting antenna such as the type or dimensions of the cables. In the current development phase, KERI has its own PA module design for ground and airborne environments and has successfully conducted WPT tests. Although the mechanical characteristics of these PA modules, such as size and weight, may differ from those required for a 27U CubeSat application, reviewing the input signal to the transmitting antenna remains valuable.

**Figure 2:**
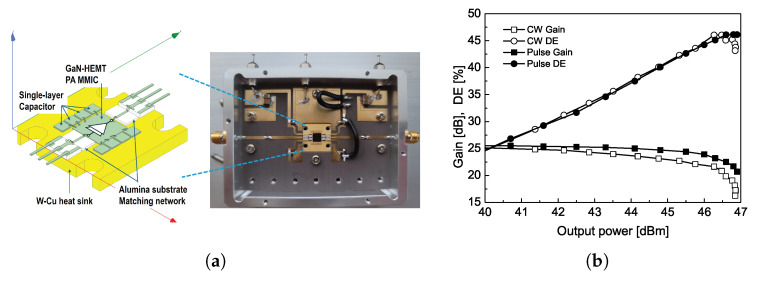
(**a**) Photograph of fabricated power amplifier module on evaluation board, and (**b**) frequency response at 5.8 GHz.

Figure 2 presents a photograph of the developed PA module on the evaluation board along with its measured output gain characteristic at the center frequency of 5.8 GHz. The two-stage GaN-HEMT MMIC PA, designed using a 0.25 μm process from WIN Semiconductor, operates at 5.8 GHz with a fully matched 50 Ω characteristic. The alumina substrate-matching circuit is designed considering wire bonding and parasitic elements from the device assembly package. All components were extracted using HFSS 3D EM simulation and incorporated into the ADS Momentum. The measured 1-tone test results at the center frequency of 5.8 GHz are shown in Figure 2b, indicating a gain of 24.5 dB to the continuous wave (CW) or pulsed input signal with an output power of 47.6 dB. A drain efficiency (DE) of 44.9% is obtained under an operating voltage of 28 V. As depicted in the photograph of the evaluation board and its measured results, the PA module effectively generates power and has been tested as part of a WPT system in both ground and airborne environments. Furthermore, it has the potential to be further developed in the near future to meet the requirements of WPT systems for space applications. Please be aware that the detailed design schematic inside the PA module and additional measured results are not included in this manuscript as they are beyond the scope of the transmitting CP antenna design.

### 2.2. Circularly Polarized Antenna Design

#### 2.2.1. Single Radiator and Feed Line Designs

**Figure 3:**
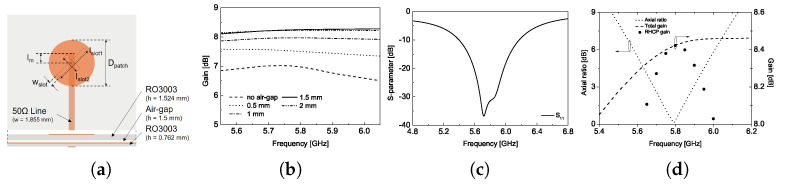
(**a**) Single radiator configuration, (**b**) gain variation with different air-gap thickness, (**c**) simulated return loss, and (**d**) simulated AR and gain. ($l_{slot1}$ = 16.2, $l_{slot2}$ = 8.5, $D_{patch}$ = 17.6, all in [mm]).

The CP array antenna is designed based on a circular microstrip patch as a single radiator, as shown in Figure 3a [12]. To enhance gain performance, an air gap is introduced between the feed line and the circular patch, improving radiation efficiency compared to previous designs [13]. This air-gap configuration minimizes substrate losses and enhances impedance matching, leading to noticeable radiation efficiency and gain improvement, as shown in Figure 3b. Based on the simulation results, an optimal air-gap thickness of 1.5 mm between the coupling slots and the radiating element is selected. This air gap is implemented using a fixture between the substrates, ensuring structural stability. The detailed dimensions of the single radiator for the simulation are given in the caption of Figure 3. Figure 3c,d present the simulated design results of the single radiator. Note that all simulation results in this manuscript were obtained using Ansys Electronics Desktop 2021 R1. The results demonstrate that the circular-shaped single radiator patch is optimally designed to operate at a center frequency of 5.8 GHz, achieving excellent impedance matching, AR, and circular polarization gain characteristic.

**Figure 4:**
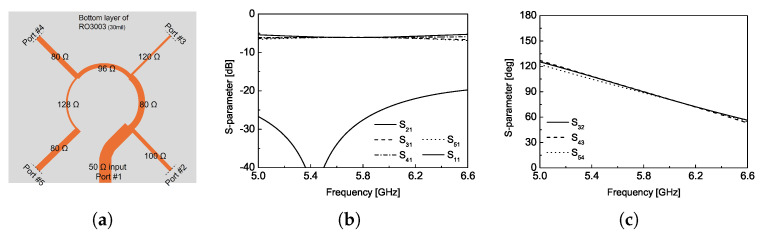
(**a**) Feed line configuration, (**b**) magnitude of simulated S-parameters, and (**c**) phase of simulated S-parameters.

Figure 4 shows a schematic of 1:4 power divider and its S-parameters simulation results. The circularly polarized wave propagation of the proposed antenna design can be achieved by determining a physical rotation, $\phi_{pm}$, and an electrical rotation, which signifies the phase change, $\phi_{em}$, when *m*-number of radiating units are implemented [14,15]. In this work, four numbers of the circular-shaped radiating unit given in Figure 3 will be attached at port 2 ∼ 5 shown in Figure 4a for the unit CP antenna design. In other words, the integer, *p*, and the number of radiating number, *M*, *M* in the following equation are 2 and 4, respectively.

(1) $$\phi_{em}=\phi_{pm}=\left(m-1\right)\frac{p\pi }{M},\;\left(1\leq m\leq M\right).$$

Figure 4b,c show the magnitude of the S-parameters and the phase differences between ports. For more details, the one-quarter of power division ratio can be obtained at each output port (port 2∼5) and the excellent input impedance-matching characteristic can also be obtained by fine tuning the width of microstrip feed line. The $90^{\circ }$ phase difference between output ports can also be precisely realized by changing the length of microstrip feed lines and its results coincide with the phase requirement for CP propagation calculated from the equation above. In summary, the detailed dimensions of the single radiator and the associated feed line for the 2 × 2 array module are provided in Table 1.

In the following section, we provide a detailed performance evaluation of the proposed CP array antennas, which are designed based on the single radiator and feed line design presented in this section. First, a single large panel is designed with an 8 × 8 array configuration, consisting of 64 radiating elements. Second, four separate units, each containing a 4 × 4 array, are also fabricated and its radiation characteristics are measured to compare with the single large-panel configuration. This comparison ensures that the proposed antenna structure maintains its performance regardless of the interface between the PA modules and the power-transmitting antenna. Lastly, two large panels, each identical to the initially designed 8 × 8 array antenna, are connected using deployable hinges. Each configuration corresponds to the mounting configurations previously described in Figure 1.

#### 2.2.2. Array Antenna Characteristics for Various Mounting Scenarios

**Figure 5:**
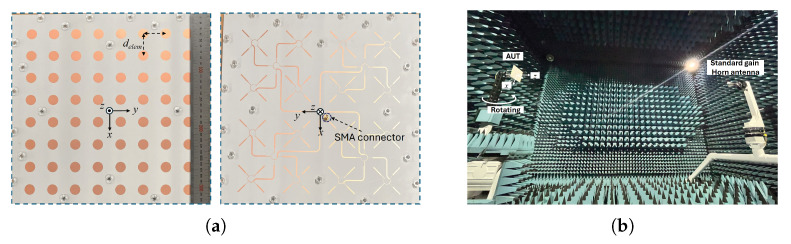
(**a**) Fabricated 8 × 8 array CP antenna for configuration ♯1 and (**b**) measurement setup in an anechoic chamber.

Figure 5 presents photographs of the fabricated CP antenna along with the measurement setup in an anechoic chamber. The circularly shaped radiating patches are arranged with equal spacing, set at a distance of 0.74λ, which corresponds to 38.25 mm in terms of the element spacing, $d_{elem}$. The specific value of the radiating element spacing is selected considering the size of a commercially available substrate suitable for the mounting configuration. In addition, the chosen spacing helps reduce mutual coupling between elements and improves sidelobe characteristics [16]. On the rear side of the proposed CP array, the feed network is implemented using microstrip lines. The fundamental structure of the feed line, originally designed to sequentially rotating radiation elements shown in Figure 4a, is expanded to accommodate all 64 elements in the array. To evaluate the radiation characteristics, the fabricated antenna is tested in an anechoic chamber using a linearly polarized standard gain horn antenna as the reference. The axial ratio and gain characteristics of the proposed antenna are determined while accounting for the polarization mismatch between the antenna under test (AUT) and the horn antenna. A standard gain horn antenna (QRH-002G-018G, MTG, Chungnam, Republic of Korea), operating over the 2–18 GHz frequency range, is employed for the radiation pattern measurements. The anechoic chamber used for the measurement has dimensions of 11 m × 8 m × 8 m, which are sufficient for accurate measurement of the radiation patterns at the center frequency of 5.8 GHz.

**Figure 6:**
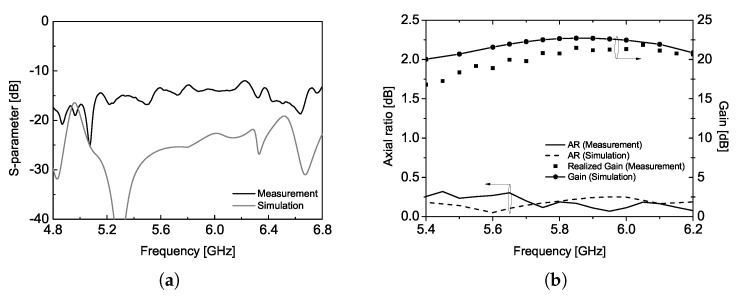
Comparison of the simulation and measurement results, (**a**) $S_{11}$ and (**b**) axial ratio and gain.

The proposed CP array antenna achieves a gain larger than 20 dBi at the center frequency of 5.8 GHz while demonstrating excellent return loss and axial ratio characteristics over a broad bandwidth. This radiation characteristic is attributed to the unique sequentially rotated feed line, which effectively cancels out reflections near the center frequency at the input port, thereby enhancing the operational bandwidth, as illustrated in Figure 6. The simulation results agree well with the measured data, validating the proposed antenna design. Note that the return loss was measured using Keysight PNA E8363B.

**Figure 7:**
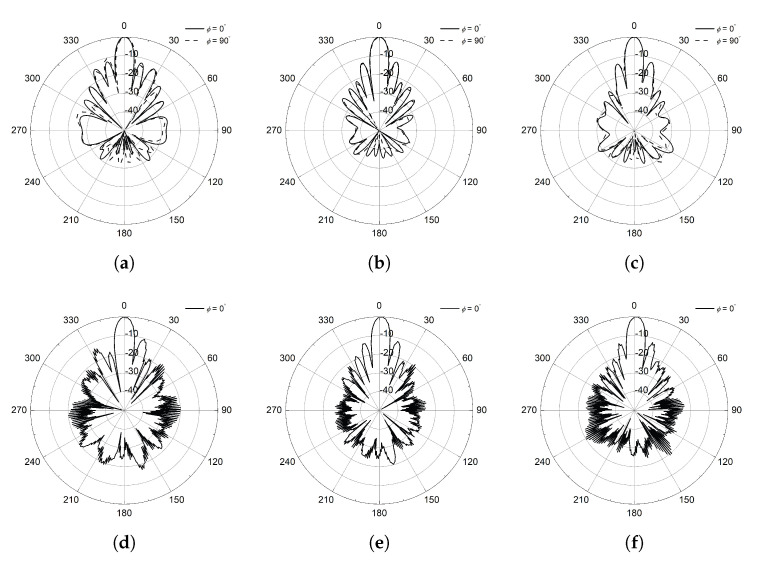
Simulated CP radiation patterns at (**a**) 5.45 GHz, (**b**) 5.8 GHz, (**c**) 6.15 GHz; measured radiation patterns at (**d**) 5.45 GHz, (**e**) 5.8 GHz, and (**f**) 6.15 GHz.

The simulated and measured radiation patterns at various frequency points are shown in Figure 7. The simulation tool provides the ideal CP radiation patterns and plotted at 5.45, 5.8, and 6.15 GHz. Both ends of the frequency points are selected, for which the sidelobe level is 10 dB smaller than the peak of the pattern and corresponds to about 12% bandwidth. The half-power beamwidth varies between $8^{\circ }$ and $9^{\circ }$ in the operational bandwidth with an extremely small axial ratio less than 0.3 dB. The radiation pattern of the proposed CP antenna is measured using a conventional linearly polarized standard gain horn antenna and the spinning radiation patterns are obtained [17]. Measurement results shown in Figure 7d–f provide the agreeable characteristics in terms of the sidelobe level and half-power beamwidth with the simulation results. Please note that the radiation patterns in $\phi =90^{\circ }$ are not provided in the measurement results due to the symmetric structure of the proposed CP antenna design. In addition, all radiation patterns presented in this study are normalized to the peak gain values at each frequency to compare the radiation characteristics such as sidelobe level and axial ratio with ease. These experimental results given in Figure 6 and Figure 7 can be a reference of the transmitting antenna for WPT application in our study, and it corresponds to the first mounting configuration.

**Figure 8:**
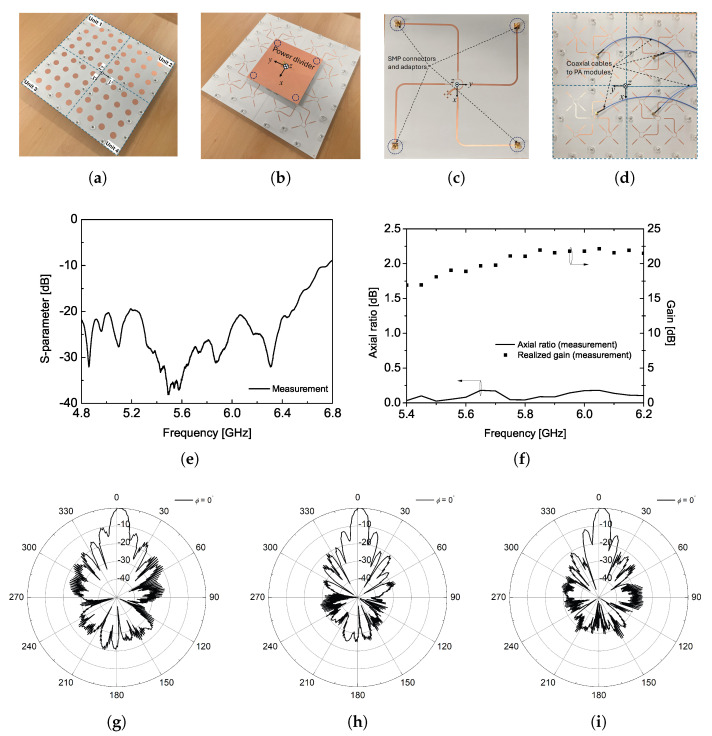
(**a**) Fabricated 4 units of 4 × 4 CP antenna for configuration ♯2 (front view), (**b**) rear view of the fabricated antenna with power divider, (**c**) front view of the power divider, (**d**) coaxial cable connection example, measured (**e**) $S_{11}$, (**f**) axial ratio and realized gain, and radiation patterns at (**g**) 5.45 GHz, (**h**) 5.8 GHz, and (**i**) 6.15 GHz.

The first mounting configuration of the proposed CP antenna, designed with a large panel, is divided into four units and each unit contains a 4 × 4 CP array antenna with a separate feed point. The fabricated four units of the CP antenna are shown in Figure 8a, where the units are seamlessly combined to form a large panel which is of identical size with the one from the first configuration. To effectively measure the radiation pattern in the anechoic chamber, an additional 1:4 power divider was designed and fabricated to feed the four input ports using a coaxial cable. This power divider is attached to the rear side of the four units, as depicted in Figure 8b, with its detailed design provided in Figure 8c. This configuration corresponds to the second mounting configuration described in Figure 1, demonstrating that the proposed CP array antenna can be implemented regardless of the interface between the PA modules and the transmitting antenna. In other words, we would like to verify that the proposed CP antenna design, with four separate feed lines, can replace a single large-panel antenna design when WPT system specifications such as efficiency, output power level, or the number of PA modules vary to meet different application requirements. Furthermore, this second mounting configuration is applicable to 27U CubeSats, where multiple separate PA modules interface with the antenna using four coaxial cables, as shown in Figure 8d as an example. Figure 8e–i present the measured CP antenna performance, including return loss, gain, axial ratio, and radiation patterns. For more details, the return loss of the fabricated four-unit CP antenna remains below −20 dB at the center frequency of 5.8 GHz, even when measured using a 1:4 power divider along with four pairs of additional SMP connectors and bullets. These SMP connectors and bullets are commercially available from Amphenol (SMP-MSLD series). The radiation characteristics of the fabricated CP antenna, including the gain variation trend with frequency, excellent axial ratio (AR), half-power beamwidth, and sidelobe level, closely resemble those of the CP array antenna designed on a large single panel. These results confirm that the proposed antenna design effectively maintains its radiation performance as a transmitting antenna in the WPT system, regardless of the interface between the PA modules and the transmitting antenna.

**Figure 9:**
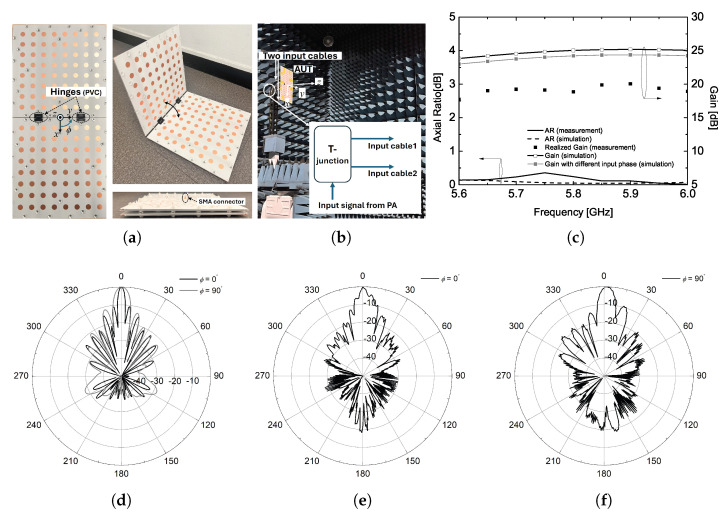
(**a**) Two CP antennas with deployable hinges for configuration ♯3, (**b**) measurement setup, (**c**) simulated and measured AR and gain, (**d**) simulated radiation pattern, (**e**) measured radiation pattern (ϕ = $0^{\circ }$), (**f**) measured radiation pattern (ϕ = $90^{\circ }$).

To investigate the radiation characteristics of the transmitting antenna in the third mounting configuration, two large panels are connected using deployable hinges allowing for stowed or deployed configurations. This mechanical operation is feasible because the input SMA connectors for each panel are placed in the same plane when the panels are fully deployed, as illustrated in Figure 9a. In an anechoic chamber, the input signal from a signal generator is fed into the fabricated antenna through a T-junction and two identical coaxial cables, as depicted in the inset of Figure 9b. Simulation results indicate that in the ϕ = $0^{\circ }$ plane, the gain increases by 3 dB, and the beamwidth is reduced by half, while the beamwidth in the other plane remains unchanged. It should be noted that the radiation pattern for the third configuration is plotted only at the center frequency of interest, as this frequency is of primary importance for the applicable WPT system.

As shown in the measurement results, the antenna gain with two panels does not reach the values predicted by simulations. From the measured radiation pattern in the ϕ = $0^{\circ }$ plane, it is observed that the first sidelobe unexpectedly shifts too close to the main lobe. This results in a broadened main lobe and the elimination of the first null. To address this issue, further optimization of the radiation patch arrangement along the x-axis is required when connecting the two panels using deployable hinges. Key factors to consider include element spacing, hinge size, and the gap between the two panels. Another important consideration is the input phase response at each feed point. As depicted in the inset of Figure 9b, each 8 × 8 array antenna is fed using two input coaxial cables during the measurement.

**Figure 10:**
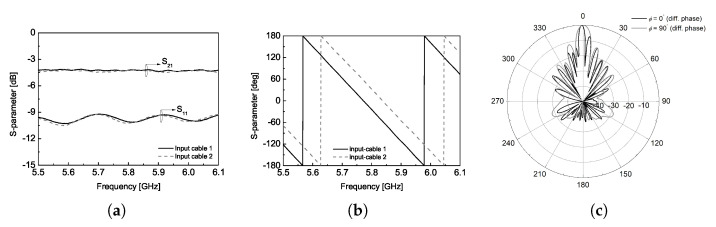
(**a**) Measured S-parameter magnitude of T-junction with input cables, (**b**) measured $S_{21}$ phase of T-junction with input cables, (**c**) simulated radiation pattern with different input phase.

The measured S-parameters of the T-junction with two input cables are provided in Figure 10. As shown in Figure 10a, there is minimal magnitude imbalance between the two cables. However, an additional loss of approximately 1 dB is introduced by the cables, and their impedance-matching performance is not optimal. More importantly, a phase imbalance was observed between the two cables, causing a deviation of approximately $50^{\circ }$ between the input signals to the two panels at the center frequency. To evaluate the impact of this phase mismatch, the radiation pattern of the two-panel CP antenna was simulated with different phase excitation. Figure 10c presents the results when the two input signals have a $50^{\circ }$ phase difference. The simulated radiation pattern shows an obvious tilt in the peak of the ϕ = $0^{\circ }$ plane, along with an increase of approximately 3.25 dB in the first sidelobe level. In addition, this phase imbalance may cause an additional degradation in the antenna gain performance, as shown using the solid gray line in Figure 9c.

## 3. Discussion

To achieve the desired radiation characteristics of a transmitting CP array antenna with multiple input signals, phase imbalance must be carefully managed. The radiation characteristics of the four-unit CP antenna can be effectively realized when the input phases are nearly identical to those obtained from a single large-panel design utilizing a 1:4 power divider. Specifically, the phase difference between the four output signals from the power divider is approximately $90^{\circ }$, with equally divided magnitude, making it suitable for generating CP antenna performance. Additionally, as the transmitting units are directly connected to the PA module via coaxial cables, as shown in Figure 8d, the PA modules must be capable of adjusting their output phase responses to maintain the performance of the transmitting antenna. Furthermore, in a WPT system where two large panels are connected using deployable hinges, as shown in Figure 9a, to enhance the radiation pattern and gain, careful consideration must be given to the phase response of the feedline. Ensuring that both panels receive electrically identical input signals is crucial; otherwise, the CP array antenna may not achieve the expected performance improvements. The characteristics of the proposed CP array antenna with various mounting configurations are summarized and compared with those reported in the literature in Table 2. The proposed 8 × 8 CP array antenna, especially the first mounting configuration, can be fairly compared with the previously published ones. Owing to the unique feature of the sequentially rotated feed network, the proposed antenna, which employs a single-panel structure with simple microstrip patch radiators, achieves a wide AR bandwidth. Furthermore, its peak gain at the center frequency is comparable to that of state-of-the-art CP antennas. It should also be noted that the use of a linearly polarized standard gain horn antenna for the radiation pattern measurement in the anechoic chamber introduces an inconsistency in gain units reported in Table 2. Nonetheless, the fabricated CP antenna shows a reasonable and competitive gain performance at the center frequency.

## 4. Conclusions

We propose an 8 × 8 circularly polarized (CP) array antenna design constructed using circular-shaped microstrip patches. A single radiating element, designed to operate at 5.8 GHz, generates CP radiation characteristics through coupling between the feed line and the patch via a cross-shaped slot. Three possible mounting configurations of the transmitting antenna, as part of a wireless power transmission (WPT) system likely operating in space with a 27U CubeSat, are considered. The proposed CP array antennas are fabricated, and their performance is evaluated. Measurement results indicate that the CP antenna on a single large panel achieves a return loss of less than −10 dB, an antenna gain exceeding 20 dB, and an excellent axial ratio of less than 0.5 dB at the center frequency with a half-power beamwidth of approximately $8^{\circ }$. Furthermore, it is confirmed that the radiation characteristics remain consistent even when the large panel is divided into four units. To assess performance under different mounting configurations, two large panels are connected using deployable hinges. Measurement results demonstrate that the axial ratio and gain characteristics remain comparable across different configurations. To prevent undesired gain reduction or asymmetrical sidelobe levels, the phase response of the input signals to the CP antennas forming the transmitting antenna must be carefully managed.

## Figures and Tables

**Table 1:** Detailed dimensions of the single radiator and the feed line structure.

Description	Patch diameter, $D_{patch}$	Slot length, $l_{slot1}$	Slot length, $l_{slot2}$	Slot width, $w_{slot}$	Microstrip line for matching $l_{m}$
Value	17.6 mm	16.2 mm	8.5 mm	1.5 mm	4.5 mm
Description	80 Ω line width	96 Ω line width	100 Ω line width	120 Ω line width	128 Ω line width
Value	0.838 mm	0.570 mm	0.514 mm	0.287 mm	0.270 mm

**Table 2:** Comparison table.

	No. Radiating Elements	Center Frequency, $f_{0}$ [GHz]	Antenna Size	Peak Gain at $f_{0}$	Measured Impedance BW [GHz]	Measured Axial Ratio BW [GHz]
[6]	2 × 2	3.5	2.41 × 2.41 × 0.062 [$\lambda^{3}$]	16.3 dBic	2.7–4.1	2.82–3.88 (31.6%)
[7]	4 × 4	66	4.42 × 8.48 [$\lambda^{2}$]	20.5 dBic	55.83–71.48	57–73 (23.8%)
[8]	4 × 4	60	5.46 × 5.48 × 0.51 [$\lambda^{3}$]	19.3 dBic	54.6–67.3	57–67 (21.5%)
[9]	1 × 4	4.7	0.99 × 0.42 × 0.3 [$\lambda^{3}$]	14 dBic	3.4–6.5	4–6 (42.0%)
[10]	2 × 2	5.8	1.82 × 1.87 [$\lambda^{2}$]	10.24 dBi	5.078–6.080	5.08–6.08 (18%)
This work (conf.1)	8 × 8	5.8	10.89 × 10.89 × 0.13 [$\lambda^{3}$]	22.65 dBi	4.06–7.47	3.80–7.95 (71.6%)
This work (conf.2)	8 × 8	5.8	10.89 × 10.89 × 0.13 [$\lambda^{3}$]	21.06 dBi	4.01–7.25	4.30–8.85 (78.4%)
This work (conf.3)	8 × 16	5.8	10.89 × 22.28 × 0.13 [$\lambda^{3}$]	19.10 dBi	4.06–7.47	4.4–7.4 (51.7%)

## Data Availability

The data that support the finding of this study are available within the article.
